# A Framework for an Indoor Safety Management System Based on Digital Twin

**DOI:** 10.3390/s20205771

**Published:** 2020-10-12

**Authors:** Zhansheng Liu, Anshan Zhang, Wensi Wang

**Affiliations:** 1 College of Architecture, Civil and Transportation Engineering, Beijing University of Technology, Beijing 100124, China; zhanganshan@emails.bjut.edu.cn; 2 Key Laboratory of Urban Security and Disaster Engineering of Ministry of Education, Beijing University of Technology, Beijing 100124, China; 3 Faculty of Information Technology, Beijing University of Technology, Beijing 100124, China; wensi.wang@bjut.edu.cn

**Keywords:** digital twin, Internet of Things, support vector machines, building information modelling, indoor safety management system

## Abstract

With the development of the next generation of information technology, an increasing amount of attention is being paid to smart residential spaces, including smart cities, smart buildings, and smart homes. Building indoor safety intelligence is an important research topic. However, current indoor safety management methods cannot comprehensively analyse safety data, owing to a poor combination of safety management and building information. Additionally, the judgement of danger depends significantly on the experience of the safety management staff. In this study, digital twins (DTs) are introduced to building indoor safety management. A framework for an indoor safety management system based on DT is proposed which exploits the Internet of Things (IoT), building information modelling (BIM), the Internet, and support vector machines (SVMs) to improve the level of intelligence for building indoor safety management. A DT model (DTM) is developed using BIM integrated with operation information collected by IoT sensors. The trained SVM model is used to automatically obtain the types and levels of danger by processing the data in the DTM. The Internet is a medium for interactions between people and systems. A building in the bobsleigh and sled stadium for the Beijing Winter Olympics is considered as an example; the proposed system realises the functions of the scene display of the operation status, danger warning and positioning, danger classification and level assessment, and danger handling suggestions.

## 1. Introduction

With the rapid digital revolution in the 21st century, an increasing amount of information technology has been applied to human residential space to improve its comfort, energy saving, and safety. The concepts of smart cities [1], smart buildings [2], and smart homes [3] are being increasingly researched. These studies are mainly focused on environmental, energy, and safety issues [4,5,6,7]. Safety is an important research direction for realising smart living spaces with real-time monitoring, real-time interaction, and automation [6]. To achieve these functions, many studies have been performed on the use of the Internet of Things (IoT), the Internet, and machine learning in safety management [2,8,9]. However, at present, safety systems are independent and cannot realise integrated information analysis or the automatic auxiliary processing of danger. Additionally, the overall connection between the safety system and the building information is poor. Furthermore, the safety management staff cannot quickly formulate a targeted treatment strategy in the case of danger, because they cannot quickly and intuitively understand the indoor layout around the location with danger. Thus, the integration and interaction of buildings and safety systems is necessary.

With the development of information technology, data have become an important factor in production. The use of data for guiding the development of various industries has become an inevitable trend. In this context, the digital twin (DT) is attracting increasing attention. The DT involves the creation of a virtual object in the digital world that corresponds to a physical object. The performance improvement and capacity expansion of physical entities can be realised through data fusion analysis, interactive feedback, and iterative optimisation between physical and virtual objects. The implementation requires the integration of multidisciplinary technologies. The DT fully exploits the information in the virtual object to provide efficient, real-time, and intelligent services for the physical world [10,11]. Considerable research has been performed on using DTs to realise the entire lifecycle service of industrial products [12,13].

DT can be realised through various technologies, such as the IoT [14], data/control models [15], and machine learning [16]. The role of the DT is to provide feedback for real-world physical systems to improve the performance of the systems. A DTM supported by appropriate algorithms can make relevant conditions correspond to the performance in the real physical world, and then propose improvements to the systems in the real world. Such methods have been applied for the evaluation of the structural health of buildings [17] and the prediction of equipment failure [18].

Safety is an important component of the performance of a building. The current safety management systems only focus on the collection of building indoor information, and cannot be fully integrated with building information to give danger-handling suggestions. Combining the IoT and BIM to form a DTM and then matching it with algorithms to improve the safety performance of the building is a direction that can be considered. However, there has been little research on the application of DTs to the indoor safety management of buildings.

The main difficulties of applying DTs in indoor safety management are as follows: (1) There is no mature method for combining the building information with dynamic safety information. (2) At present, safety management systems are independent, and the method for the integrated analysis of building safety information is not mature. The method proposed in this paper realises the real-time collection and integrated management of indoor safety data by combining BIM with the IoT. Additionally, the scene management of indoor safety data is realised by using the three-dimensional (3D) visual function of BIM. To investigate the method of the comprehensive analysis of complex safety data, the feasibility of using a support vector machine (SVM) to analyse safety data for realising the automatic classification and evaluation of danger is verified.

Herein, an indoor safety management system (ISMS) based on the DT method is proposed. In the system, the IoT is used to collect indoor safety data. An SVM is used to automatically classify and evaluate the danger level of indoor safety data, and it was validated. Additionally, this paper proposes displaying the collected safety data and types and levels of danger on an intelligent safety management platform integrated with BIM. This scene display method allows safety management staff to intuitively and quickly understand accidents.

## 2. Literature Review

### 2.1. Artificial Intelligence of Things (AIoT)

With the development of information technology, IoT technologies are playing increasingly important roles in various industries [19]. IoT technologies, including sensors, robots, network facilities, and intelligent devices, are innovated and developed to satisfy the needs of various industries [20]. There have been many such innovations with regard to safety in the construction operation stage. In [21], indoor positioning technology was combined with BIM, and an environment-aware beacon deployment algorithm based on positioning and BIM information was designed to improve the indoor positioning accuracy and reduce the work required to deploy sensor networks, which improves the robustness of sensor networks in buildings in emergencies. In [22], BIM was combined with wireless sensor networks to integrate personal positioning information, Bluetooth-based evacuation/rescue route optimisation information, and mobile navigation equipment information, yielding an intelligent, two-way fire disaster-prevention system framework. Additionally, the mining of large amounts of data collected by the IoT using artificial intelligence (AI) to integrate AI and the IoT for obtaining the Artificial Intelligence of Things (AIoT), which enhances the intelligence level of the residential space, is an important research topic. In [8], an SVM was used to mine data for realising the automatic and rapid judgement of the indoor danger level on the basis of the collection of indoor environmental information by the IoT. In [23], an intelligent disaster-prevention hard hat was developed according to the concept of the AIoT to improve the safety of personnel in high-risk rescue sites and home environments. In [24], machine learning algorithms were used to automatically classify the indoor environment based on the indoor environment data collected by the Internet of Things.

### 2.2. Dynamic BIM

BIM technology has been widely used in building design, construction, and operation management. It can not only provide an intuitive 3D visual model of buildings but also realise the information storage and management of the entire lifecycle of buildings. BIM is widely used in the design and construction stage of the construction industry. Therefore, buildings are completed with the formation of BIM models. These models can be directly applied to solve the problems in the operation stage. It is no longer necessary to build a new database or 3D model for the operation stage [25]. Therefore, BIM is ideal for scene-based building safety management. In general, BIM can provide an information hub for building safety management, but it is a static hub which cannot realise the real-time automatic updating of building safety management information. For realising the real-time updating of information, the combination of BIM and the IoT is a feasible solution. Studies have increasingly combined the two to solve problems in the architecture, engineering, and construction (AEC) industry [26]. For example, in [27,28], a dynamic 3D visualisation method was presented for dealing with fire danger by combining an indoor positioning system with BIM, which improved the emergency-handling capacity of buildings. In [29], a visual and persuasive energy-saving system was constructed by combining the IoT with BIM to improve the comfort degree of the indoor space and the energy-saving performance. In [30], the construction level of prefabricated components was improved by combining long-range radio (LoRa) technology with a cloud-based BIM model. In [31], machine-learning algorithms were used to comprehensively analyse the data from BIM and the data collected by the IoT for monitoring and predicting the operating status of construction equipment; this method realised the early warning of equipment operation faults and proposed equipment replacement and maintenance schemes predictably.

### 2.3. DTs

The concept of DTs was first used by National Aeronautics and Space Administration (NASA) and the United States Air Force (USAF) to predict the residual life and health maintenance of spacecraft [32]. The use of DTs introduces a new approach for synchronising the real physical world with the virtual digital world. The development of emerging information technology allows DTs to be applied in various industries, including aviation [33], energy [34], mining [35], shipping [36], and transportation [37]. To promote the application of DTs in related fields, according to the 3D model of DTs proposed by Professor Grieves a five-dimensional model of DT was proposed. Ten major potential application fields for the model were discussed. Besides this, construction and safety were also considered as potential application areas for digital twins [38]. As a technique for realising a cyber–physical system [38,39], the DT method integrates multiple new information technologies such as the Internet, the IoT, artificial intelligence, and digital modelling to achieve interaction between the real physical world and the virtual digital world, which helps systems in various industries to achieve high-level intelligence and automation [40]. In [41], the applications of BIM were reviewed to pave the way for construction DT, and the areas for future research in the AEC industry were elaborated. At present, digital twins have been applied in the field of smart cities [42], building construction, maintenance, and strengthening operations [43,44]. Furthermore, there have been studies using the combination of the BIM model and the IoT to construct a digital twin model of the building [17,18].

### 2.4. Research Gaps and Novelty

Although there are many information-technology applications in the field of indoor safety [45], few studies have been performed on the application of DT technology in the field of indoor safety. Moreover, because of the independence of the safety systems in the building, it is impossible to conduct a comprehensive automatic analysis of various dangers. Additionally, the geolocation of the danger remains at the level of text or two-dimensional drawings, because the safety system cannot be combined with the 3D layout of the building; thus, the building environment in which the danger is located cannot be directly displayed. To solve these problems, in the present study BIM, the IoT, and the Internet were used to build a DT model for indoor safety. The model was employed to conduct meaningful investigations as follows: (1) According to the indoor safety data collected by the IoT, the comprehensive data management of different types of dangers was realised. (2) The feasibility of automatic danger classification and level evaluation using SVM was proven. (3) According to the comprehensive data analysis of safety, this study proposes a method for automatically generating danger-handling suggestions to guide safety management staff in dealing with indoor dangers. (4) The 3D scene management of indoor safety is realised to help the safety managers quickly understand the indoor layout around the location with danger. This innovative method, which integrates technologies such as the IoT, the Internet, machine learning, and BIM, improves the management level of indoor safety and is of great significance for future research.

## 3. Materials and Methods

### 3.1. Concept of ISMS Integrating with DT Model

**Figure 1:**
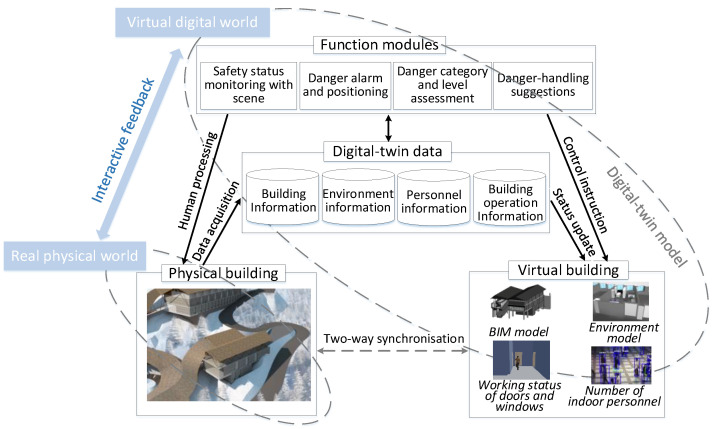
Concept of an indoor safety management system integrating with the digital twin model.

To solve the aforementioned problems, the concept of indoor safety management based on DTs is proposed. Here, the exchange of data—including building layout information, indoor environment information, indoor personnel information, and building operation information—between physical buildings and virtual buildings through perception and simulation is realised. This information characterises the safety situation of real physical buildings synchronously in the virtual digital world. In the virtual digital world, algorithms are used to analyse and process data for realising functions such as the visual data management of physical buildings, visualised building danger alarm and positioning, danger classification and level assessment, and danger response suggestions, which help safety management staff deal with danger in real physical buildings. In this concept, the improvement of the real-world safety situation is realised with the help of the DTM in the virtual digital world. The concept is illustrated in Figure 1.

### 3.2. Methodology and System Framework

**Figure 2:**
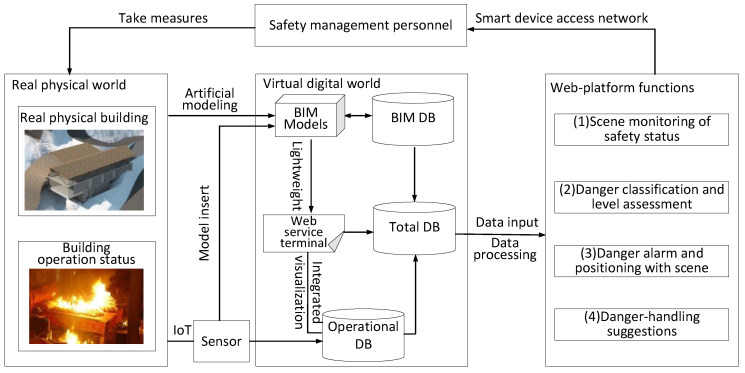
Framework of the proposed ISMS based on the digital twin method.

According to the proposed concept, a systematic framework for the development of an ISMS based on the DT method is proposed, as shown in Figure 2. In this framework, the BIM model is developed by engineers during the construction process. The operation information of the building is collected by the IoT, and the sensors in the IoT are included in the BIM model. The light-weighted BIM model is carried on the webpage so that the 3D visualisation of the building model and sensor model is realised on the webpage. Additionally, the operating data of the building collected by the sensor are transmitted to the webpage to realise data visualisation. Then, data-processing methods such as machine learning are used to analyse the data on the webpage. Finally, visual safety status monitoring, danger alarm and positioning, danger classification and level assessment, and danger response suggestions are realised on the web platform to assist the safety management staff in taking danger-handling measures. The safety management staff access the network using computers, smartphones, tablets, and other devices to obtain information and decision-making suggestions, which are helpful for dealing with dangerous events in the real world.

### 3.3. Establishment of DTM for Indoor Safety

#### 3.3.1. Information Needed to Characterise DTM

In the management system framework, a DTM corresponding to a real physical building must be built in the virtual digital world through modelling by engineers, the IoT, Internet, etc. In order to achieve the scene monitoring of the safety status and danger positioning with the scene, the 3D geometric information of buildings must be depicted. The information on the building materials, material manufacturers, and other aspects does not need to be depicted. Additionally, the DTM must contain information generated during building operation, including the indoor temperature, oxygen concentration, carbon-monoxide concentration, smoke concentration, opening and closing statuses of doors and windows, number of indoor personnel, and time.

#### 3.3.2. Processing of BIM Model

**Figure 3:**
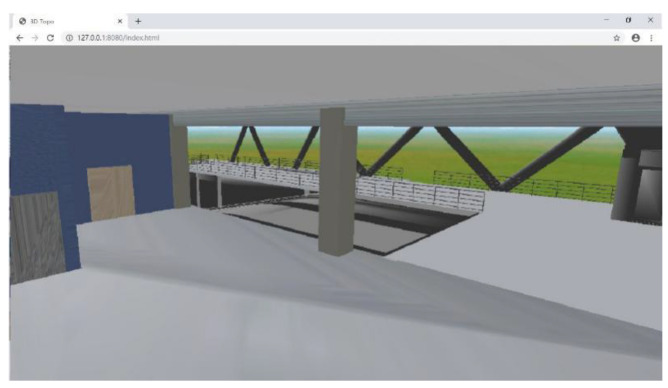
3D visualisation of the building layout on the website.

To realise the flexible access of the system using different terminals, including personal computers, tablets, and smartphones, a B/S structure was used for the proposed system. The BIM model must be uploaded so that users can access it on the webpage via the Internet and browse the indoor layouts of buildings. The BIM model of a building contains many types of professional information, such as geometric information, structural information, equipment information, and material information. The objective of this study was to use a BIM model for building layout information. It does not need structural, electrical, or other such information; therefore, this information was excluded, and only the building geometric information was retained in the BIM model. Additionally, the model was exported into an IFC file. Then, the file was read in a JavaScript environment to realise a light-weighted BIM model for improving the loading and running speeds of the BIM model on the webpage. The lightweight BIM model was loaded on the webpage through WebGL technology, which realised the 3D visualisation of the building layout and provided a 3D scene for the safety monitoring and danger positioning. The 3D visual effect of the building layout on the webpage is shown in Figure 3.

#### 3.3.3. Construction of IoT Structure

**Figure 4:**
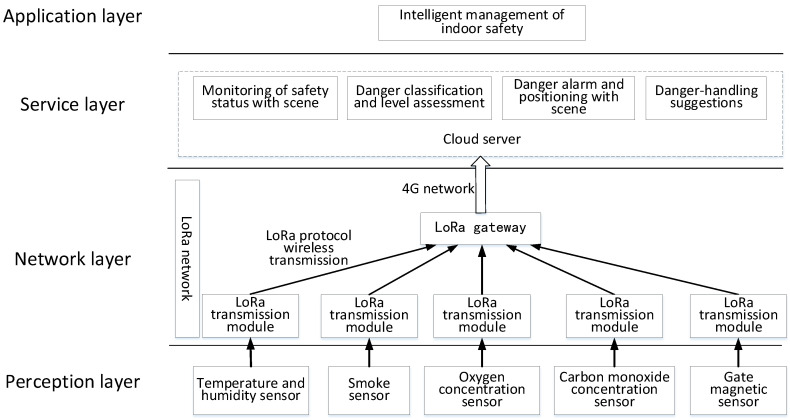
IoT structure of the indoor safety management system based on LoRa technology.

For the collection of operating data, this paper proposes an IoT system which comprises a perception layer, a transport layer, a service layer, and an application layer, as shown in Figure 4. This study uses Low-Power Wide-Area Network (LPWAN) to build an IoT system. LPWAN is a form of IoT with a lower power consumption and wider transmission range than the traditional IoT. In this study, LoRa technology was used in the LPWAN, which is essentially a spread spectrum modulation technology. Spread spectrum modulation technology has been widely used in the military and aviation fields, and LoRa technology is a low-cost wireless communication solution for manufacturing and other civil fields. In China, LoRa works in the 470/510-MHz ISM bands and can achieve long-distance coverage, with bit rates ranging from 0.37 to 46.9 kbps [30].

The perception layer includes sensors that are used to measure the operating conditions. The network layer is constructed using LoRa wireless communication technology in an LPWAN, which is used for data transmission. The service layer uses Structured Query Language (SQL) to build a database on the web and uses an SVM algorithm for data analysis and processing to realise safety status monitoring and danger alarms, danger categorisation and classification, and other functions, as well as assisting in the intelligent management of safety. With the help of these functions, the safety management staff can take action to handle danger; thus, the intelligent management of indoor safety in the application layer is realised.

(1) Perception layer.

**Figure 5:**
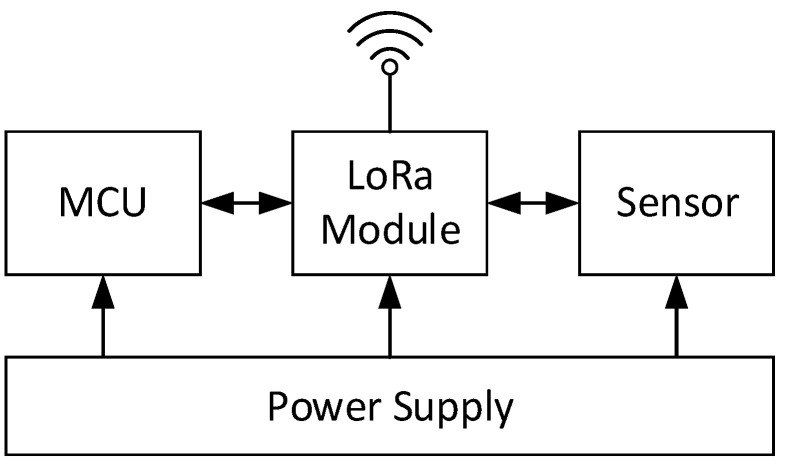
Internal structure of the sensor.

**Figure 6:**
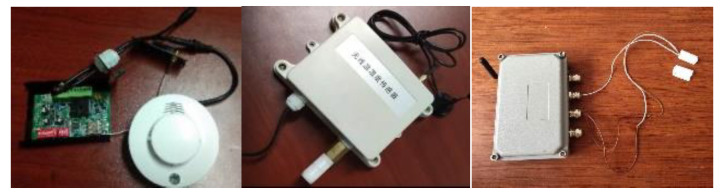
Sensing terminals.

**Figure 7:**
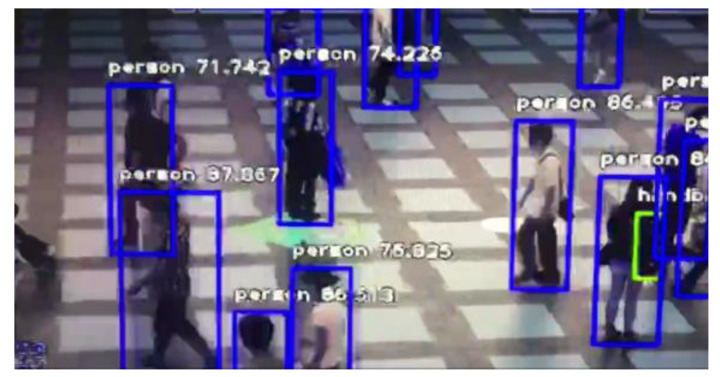
Automatic determination of the number of people using image-recognition technology.

For the perception layer, indoor environment information acquisition terminals based on the LoRa transmission protocol were developed. The terminal consists of various sensor modules, a LoRa module, a microcontroller unit control module, and a power module. Its structure is shown in Figure 5. According to the needs of indoor information collection, five types of sensing terminals were developed for sensing the oxygen concentration, carbon-monoxide concentration, smoke concentration, temperature, and opening and closing statuses of doors and windows. The sensor terminals can realise the real-time measurement of the indoor operating information of the building and wirelessly transmit this information to the LoRa gateway. The sensor parameters selected in this study are presented in Table 1. The terminal that senses the opening and closing of the doors and windows employs changes in potential. The structure of the sensing terminal is shown in Figure 5. Some of the developed sensor terminals are shown in Figure 6. In addition to using and developing LoRa-based sensing terminals, the perception layer employs cameras to obtain indoor images, along with image-recognition technology to automatically determine the number of people in the images, as shown in Figure 7. Additionally, to realise the visualisation and information storage of the corresponding position of the sensor terminal in the BIM model, family library models of different sensor terminals were established to realise terminal visualisation in the BIM model.

(2) Transport layer.

**Figure 8:**
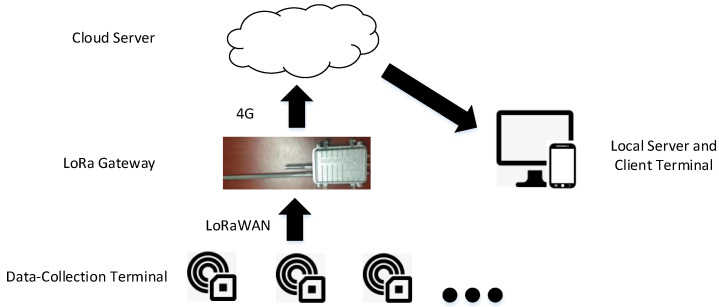
Network deployment based on LoRa technology.

A wireless transmission network based on LoRa technology was constructed. The LoRa wireless network had a star network structure. LoRa technology has the advantage of a high capacity, allowing it to realise the connection of a large number of data-collection terminals with the LoRa gateway. The sensing terminal transmits the indoor safety information obtained by the built-in sensor to the LoRa gateway through the LoRa module. The LoRa gateway then uploads the information to the cloud server through the 4G network, and the local server accesses the cloud server through the Internet to obtain the indoor safety information. A schematic of the network deployment based on LoRa technology is shown in Figure 8.

(3) Application layer.

The application layer mainly includes the following functional modules: (1) safety status monitoring with a scene, (2) danger classification and level assessment, (3) danger alarm and positioning with a scene, and (4) danger handling suggestions. Each module is described below.

(1) Safety status monitoring with a scene.

**Figure 9:**
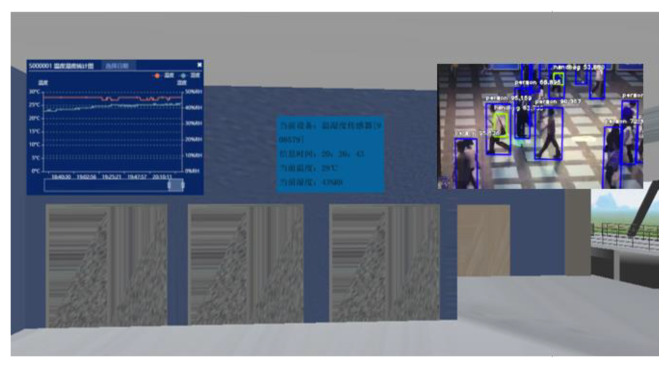
Interface rendering for safety status monitoring with a scene.

This module uploads the data collected by the sensing terminal to the webpage and associates it with the sensor. The model is associated with the camera, whereby the actual situation at the corresponding position in the building can be viewed. The perspective of the camera is fixed, while the layout of the building can be viewed from different directions through the roaming function of the BIM model on the web. When the safety management staff view different rooms from a 3D perspective, the rooms can be monitored by the cameras installed inside. Thus, virtual reality, a fixed perspective, and a mobile perspective are combined in a complementary manner. Additionally, when the model is associated with the sensing terminals, not only can the position of the terminals be observed, but also the collected data can be viewed. The data can be updated with the information collected by the sensing terminal to monitor the environmental data of the building. The interface rendering is shown in Figure 9.

(2) Danger classification and level assessment.

At present, the evaluation of the level and types of dangers mainly depends on the evaluation of safety management staff; thus, it is highly dependent on people and inefficient. This paper proposes a method where an SVM is used to analyse the collected building operating data for realising automatic danger assessment. The assessment includes the classification of danger types and the determination of danger levels. This study focused on three types of dangers—illegal invasion, overcrowding, and fire—which were divided into three levels: safe, potentially dangerous, and dangerous.

(3) Danger alarm and positioning with a scene.

To achieve the warning function for the danger victims and manufacturers, this function module is responsible for sending out alarm signals on the webpage and transmitting the signal to the alarm device in the corresponding room to sound an alarm when the room is in danger. When the BIM model of the terminal corresponding to the real position is established, a unique number is assigned to the terminal. The 3D layout of the room and the corresponding video surveillance screen can be automatically searched according to the terminal number in the room when the room is in danger to identify and locate the danger.

(4) Danger-handling suggestions.

According to the different types and levels of danger, this module provides specific suggestions for danger response, as shown in Table 2.

The system sends the danger-handling suggestions (together with the 3D scene of the location of the danger) to the safety management staff through the webpage to guide the staff in taking correct measures for dealing with the danger.

### 3.4. SVM for Intelligent Classification and Level Assessment of Danger

The relationship between danger parameters is complex and cannot be easily characterised with a reliable mathematical expression. For example, in the early stage of fire development, smoke is relatively thick, but the temperature is not high and the danger is relatively small; however, with the development of the fire, the smoke gradually decreases and the temperature increases. Moreover, many factors affect the fire parameters; thus, it is difficult to establish a reliable mathematical relationship between the types of danger, the level of danger, and the parameters.

Machine-learning methods can find complex correlations between independent variables and dependent variables through large amounts of calculations based on a series of samples, leading to reliable classification and regression. Therefore, the use of machine-learning methods to achieve the classification and rating of dangers is one of the methods that can be considered. In this study, to realise the automatic classification and level evaluation of dangers, an SVM was employed to mine the safety data collected by the IoT, and a mature SVM model was trained. Using this model, the automatic classification and level assessment of indoor dangers were realised. For each danger type, an SVM model was used to determine whether it has occurred. The danger level was evaluated using a danger coefficient predicted by an SVM model. A larger danger coefficient corresponded to a higher level of danger.

**Figure 10:**
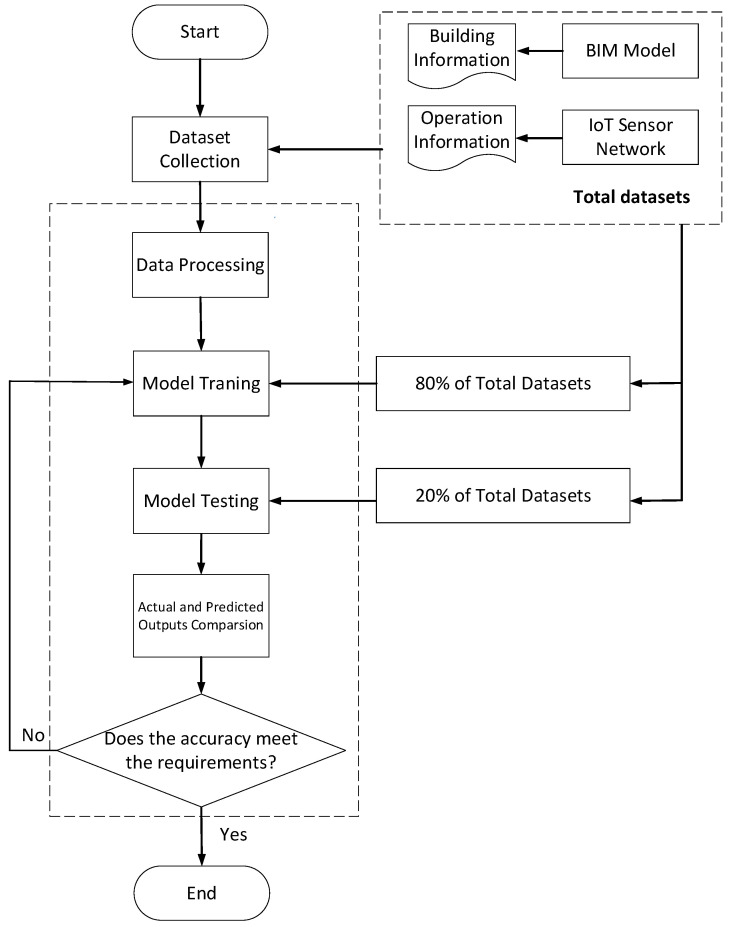
SVM prediction process.

The specific steps for the intelligent classification and level assessment of dangers are shown in Figure 10.

(1) Data collection for influencing factors.

The data used in this study for the factors influencing the danger level were from the BIM model and the operating data collected by the IoT. Information regarding the room area, room space relationship, number, and locations of doors and windows was mainly collected from the BIM model. The operating data, including information regarding the opening and closing statuses of doors and windows, temperature, carbon-monoxide concentration, oxygen concentration, smoke concentration, and number of personnel, were mainly collected by the IoT.

(2) Data processing.

The data were divided into numerical data and logical data. The logical data were quantised into numerical data. Numerical data “0” and “1” were used to represent the logical data as “false” and “true”, respectively. The time data were transformed into corresponding numerical data using Equation (1).

(1) Time = hour/24+ minute/1440 + second/3600/24.

Owing to the different dimensions of the influencing parameters, the value ranges of the transformed numerical data differed significantly. The dispersion standardisation method was used to normalise all the transformed numerical data. The transformation equation was as follows:

(2) $$x=\frac{\left(x-min\right)}{\left(max-min\right)}.$$

(3) Selection of training and test sets.

Because the number of samples with danger was significantly smaller than the number of samples without danger, the use of these data for training directly influenced the training effect of the SVM, owing to data skew. Therefore, it was necessary to pre-process the sample. The under-sampling of samples without danger was used to reduce the difference between the numbers of samples with and without danger [46]. Then, the samples without danger from under-sampling and all the samples with danger were randomly scrambled [47]. A total of 80% of these samples were randomly selected for model training, and the remaining 20% were used for model testing [48].

(4) Model training.

The key to model training is to select the proper parameters and kernel functions. In this study, the radial basis function (RBF) was selected as the kernel function because its accuracy and calculation performance are better than those of other kernel functions [49,50]. K-fold cross-validation was used to determine the kernel-function parameter and penalty coefficient.

(5) Test and effect evaluation.

Classification of danger types: The test set selected in step 3 was used to test the prediction effect of SVM. Through a comparison between the prediction results and the actual danger of classification, the prediction accuracy was calculated (using Equations (3)–(5)). A higher accuracy corresponded to the better prediction effect of the model.

**Figure 11:**
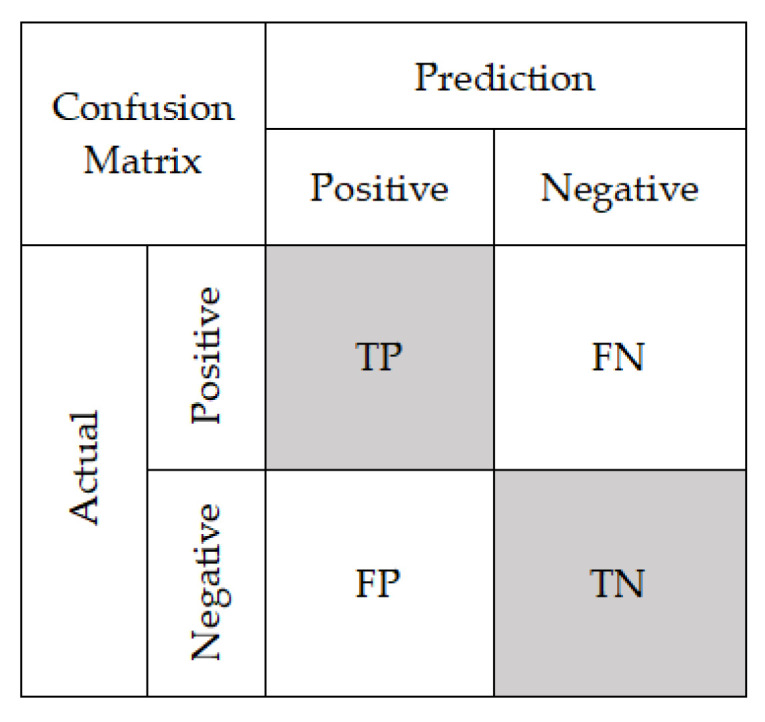
Confusion matrix.

In the prediction of the danger classification, the calculation of the classification accuracy, precision, and recall rate with the help of a confusion matrix is a common method for evaluating the classification effect. Higher values of these indicators correspond to a better classification effect [51]. The confusion matrix is shown in Figure 11, and the accuracy, precision, and recall are calculated using Equations (3)–(5).

(3) $$\mathrm{Accuracy}=\frac{TP+TN}{TP+FN+FP+TN},$$

(4) $$\mathrm{Precision}=\frac{TP}{TP+FP},$$

(5) $$\mathrm{Recall}=\frac{TP}{TP+FN}.$$

Prediction of danger level: In this study, according to the danger level of the experiment, the danger coefficient α was evaluated artificially and recorded. A larger danger factor corresponds to a higher danger level. When there is no danger, α ≤ 1; when there is potential danger, 1 < α ≤ 2; when there is danger, α > 2. The predicted coefficient is classified into the corresponding grade and compared with the actual coefficient. Consistency between the two indicates that the prediction is correct. The prediction accuracy was used to evaluate the prediction effect of the SVM model. A higher accuracy rate corresponded to a better prediction effect. Additionally, in regression prediction, the squared correlation coefficient *R*^2^ is an important indicator for evaluating the prediction effect. An *R*^2^ value closer to 1 corresponds to a better prediction effect. *R*^2^ is calculated using Equation (6).

(6) $$R^{2}=\frac{\sum_{i}{\left(y_{i}-f_{i}\right)}^{2}}{\sum_{i}{\left(y_{i}-\bar{y}\right)}^{2}},$$

where *y_i_* represents the true value, *f_i_* represents the predicted value, and $\bar{y}$ represents the average of all the true values.

When the accuracy of the classification and level division is low, step 4 and step 5 should be repeated. The parameters are adjusted until the accuracy is increased.

## 4. Case Study

### 4.1. Case Background and Scenario Simulation

**Figure 12:**
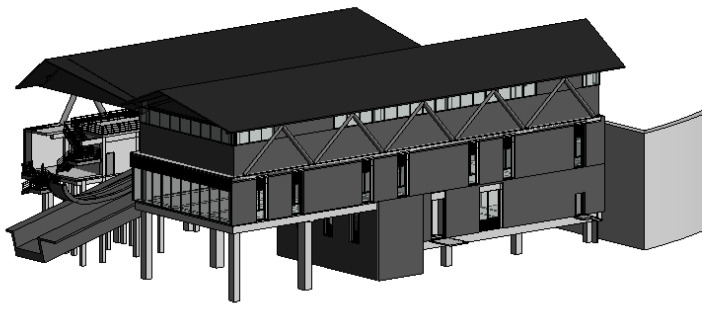
BIM model for a building in the bobsleigh and sled stadium.

A case study of a building in the bobsleigh and sled stadium for the 2022 Winter Olympic Games in Beijing is examined. The building is built on a hillside, with a total of three floors above the ground. The internal layout of the building is complex because of the complex function of the building; it serves as the starting site for the competition. There is expected to be a large flow of people and important competition supplies, such as bobsleigh sleds, which have very high safety requirements. During the design and construction stages of the stadium, a BIM model that are very consistent with the actual building was established, which contained information regarding the geometry, materials, and equipment of the building, as shown in Figure 12.

### 4.2. Implementation Process and Effect of Experiment

#### 4.2.1. Processing of BIM Model

In this study, the information in the BIM model that was not related to the building layout was removed from the model. The new BIM primitives for all types of terminals installed in the rooms were added to the BIM, at positions identical to their positions in the real world. Then, the model with terminal primitives was transformed into a lightweight model, which was implemented online using webGL technology.

#### 4.2.2. Terminal Layout

**Figure 13:**
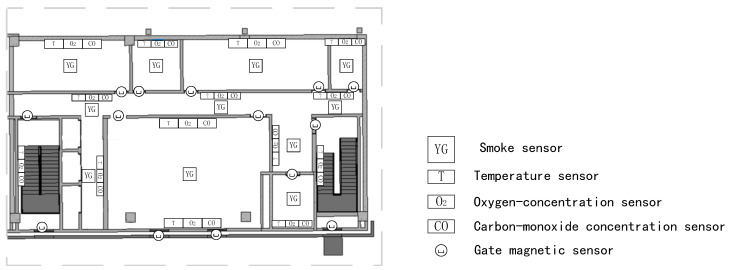
Layout of the indoor terminals.

A terminal layout method based on LoRa technology is proposed herein. Taking the second floor of the building as an example, a smoke-concentration terminal was installed at the centre of the ceiling of each room. In principle, a temperature terminal, an oxygen-concentration terminal, and a carbon-monoxide concentration terminal are installed in the central position of the wall in each room. If the room is large, two temperature terminals, oxygen-concentration terminals, and carbon-monoxide concentration terminals are installed in the room. Gate magnetic terminals for sensing the opening and closing of doors and windows are installed on each door and window of each room. The layout of some of the indoor terminals on the second floor is shown in Figure 13.

#### 4.2.3. Danger Simulation and Data Acquisition

**Figure 14:**
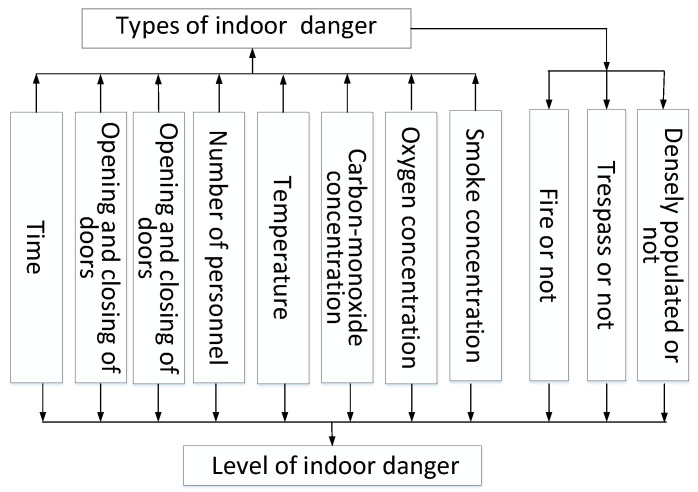
Factors affecting the indoor danger types and danger coefficient.

A stairwell was used as an example to simulate three types of danger: illegal intrusion, overcrowding, and fire. The room area was approximately 26 m^2^, and the room contained a door and a window. The eight parameters of the independent variables in the SVM used for category judgement were the time, opening and closing of the door and window, number of people, carbon-monoxide concentration, oxygen concentration, and smoke concentration. An SVM model was used for predictive training for each danger type. Each SVM model judged whether a danger occurred, so that each type of danger was considered separately when the danger occurred. After the danger-type judgement was complete, these judgements were used as new independent variables to train a new SVM for evaluating the danger level of indoor safety together with the previous parameters, as shown in Figure 14.

A room in the building was taken as an example to simulate the three danger types: illegal invasion, overcrowding, and fire. The simulation method was as follows.

(1) Illegal intrusion: The illegal-intrusion behaviour was simulated by entering the simulation room through a door or window from 20:00 p.m. to 7:00 the next day. When the people opened the door or window, the operating data collected by the IoT was marked as “illegal intrusion.” At this time, the danger coefficient of the situation was evaluated by safety management staff.

(2) Overcrowding: Many people entered the room together to simulate the overcrowding situation. Video data were collected during the simulation. The number of people in the video was automatically determined via image recognition. The experimenter judged whether there was a danger of overcrowding according to the area of the room and the number of people and recorded the judgement. At this time, the danger coefficient of the situation was scored by the safety management staff.

(3) Fire: A fire was set in the room during the experiment. The operating data were collected during the simulation and were marked as “fire.” At this time, the danger coefficient of the situation was scored by the safety management staff.

(4) Normal: The operating data collected at other times (without a danger simulation) were marked as “normal.” The danger coefficient of the situation was scored at this time.

The simulation experiment lasted 15 d. Different combustion materials were used to simulate fire 10 times, illegal invasion 13 times, and overcrowding 9 times. The data from the sensors were set to update data every 20 s. A total of 64,800 groups of operating data were collected, including 566 datasets with fire danger, 567 datasets with illegal invasion danger, and 241 datasets with overcrowding danger; the rest were normal data.

#### 4.2.4. Division Effect of Danger Types and Levels

Because the number of samples with danger was far smaller than the number of samples without danger, the training effect of the SVM was affected by data skew. Therefore, the sample information was pre-processed. The training effect was improved by under-sampling the samples without danger. All the samples with danger were combined with 1300 randomly selected samples without danger. A total of 2674 sets of samples were collected. The sample numbers for the different danger types are presented in Table 3. The data collected and quantified are presented in Table 4.

**Figure 15:**
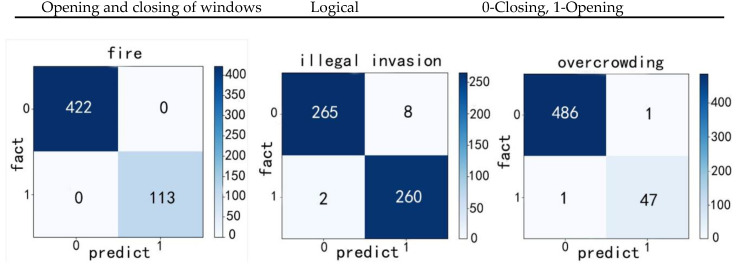
Test confusion matrices for the different danger types.

The dangers considered in this study were illegal invasion, overcrowding, and fire. An SVM model was trained for each type of danger. Then, using the method described in Section 3.2, the samples were scrambled; 80% were randomly selected for model training, and the remaining 20% were used for model testing. The test confusion matrices for different danger types are shown in Figure 15. The indices used for evaluating the classification effect are presented in Table 5. The results indicate that the SVM had a reliable effect on the classification of the danger types.

In this study, according to the danger level of the experiment, the danger coefficient α was evaluated by the safety management staff. A larger danger coefficient indicated a higher danger level. The coefficient was set as follows: no danger, α ≤ 1; potential danger, 1 < α ≤ 2; danger, α > 2. The aforementioned 2674 groups of safety data were used as independent variables, and the recorded danger coefficient was used as the dependent variable. Additionally, whether different types of similar dangers occurred according to the SVMs above was an independent variable. The number “1” indicated that the corresponding danger had occurred, and “0” indicated that the corresponding danger did not occur, as shown in Table 6. Thus, the number of independent variables was increased from 8 to 11.

**Figure 16:**
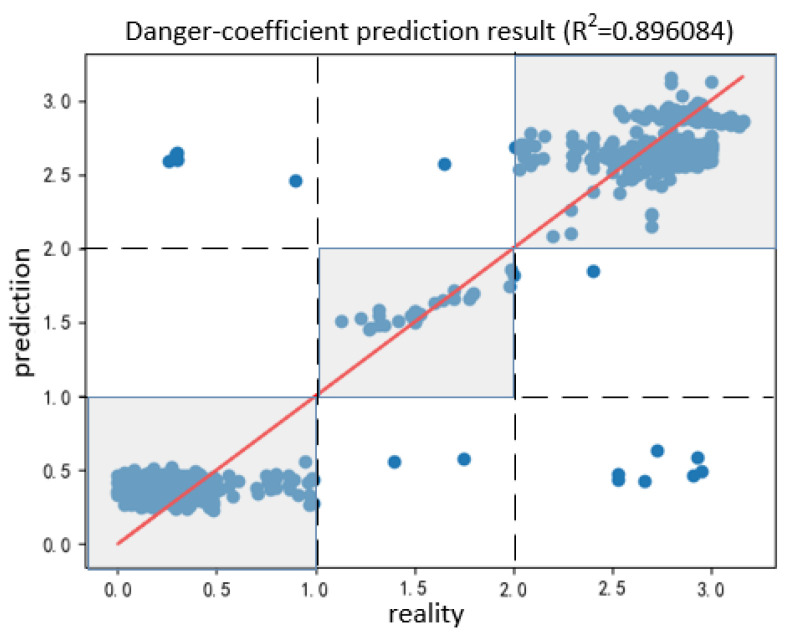
Prediction results for the danger coefficient.

The SVM was trained for danger coefficient prediction. A total of 80% and 20% of the samples were used for model training and testing, respectively. The predicted results for the safety coefficient in the test are presented in Figure 16. Here, the horizontal axis indicates the actual value, and the vertical axis indicates the predicted value. The points on the red diagonal line correspond to the predicted and actual values. The point in the shaded part corresponds to the correct prediction result of the danger level. For 535 groups of predicted data, the accuracy rate was 96.2%, and the R^2^ reached >0.89, indicating that the prediction effect of the model was good.

#### 4.2.5. Effects of Danger Alarm and Position with Scene and Handling Suggestions

**Figure 17:**
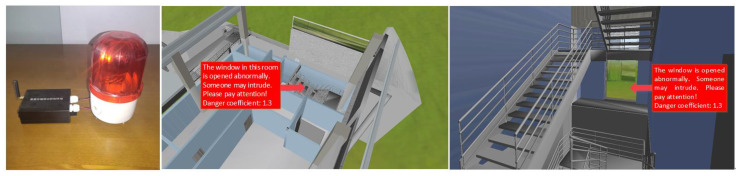
Synchronous alarms in the virtual and real worlds.

The trained SVM model was used to evaluate the real-time building operation status. The danger type and danger level of each room were automatically determined. Then, the assessment information was combined with the BIM model to display the location of the danger in three dimensions. This allowed the safety management staff to intuitively understand the scene of the dangerous location. The danger can be handled quickly and accurately because safety management staff understand the surroundings of the danger owing to the 3D indoor layout. Additionally, an alarm device in the room issued an alarm, together with an alarm on the website. The alarm device was connected to the system via LoRa technology to realise simultaneous alarms in the virtual digital world and the physical real world, as shown in Figure 17.

## 5. Discussion and Conclusions

A framework for an ISMS based on the DT method is proposed. The framework uses BIM, the IoT, and the Internet to construct a DTM for building indoor safety. BIM provides building information related to safety, information hubs, and 3D geometric models. The IoT sensors collect real-time indoor operation information and monitor the operation of indoor safety status, realising state mapping from the real physical world to the virtual digital world. An SVM performs an automatic classification and level assessment of indoor danger through the mining of safety data. The Internet provides a platform for data storage and user interaction. The method was applied to a room in a building of the bobsleigh and sled stadium for the 2022 Beijing Winter Olympic Games. The SVM was beneficial for the danger classification and level assessment, and the overall system achieved good experimental results.

The main contributions of this study are as follows.

(1) A set of methods for applying the concept of DT in the field of indoor safety was proposed. The feasibility of DTs for solving the problem of building safety was confirmed.

(2) The integration of building operating data and 3D indoor scenes was realised. The intuitiveness of the indoor safety management was improved.

(3) The feasibility of using AIoT to define the types and levels of indoor danger, which can improve the automation level and speed of the response to indoor danger, was proven.

(4) According to the classification and level of indoor danger, automatic auxiliary processing suggestions were realised. The scene data management mode was added, which relaxes the requirements regarding the quality of the safety management staff.

The proposed framework provides a good indoor safety management method which can improve the intelligence level of indoor safety management. In the future, the function of this framework will be expanded to make the suggestions for danger assistant treatment more targeted. Additionally, besides the dangers in this research, other dangers indoor environment can be taken into account based on this framework, such as the leakage of some harmful gases in the environment. Moreover, DTs will be applied to other aspects of building operation management to improve the intelligence level. For example, with reference to the framework in this article, DTMs of building structure and equipment can be established so as to improve the management of structural safety and equipment safety. This is also a direction that can be considered in the future.

## Figures and Tables

**Table 1:** Sensor parameters.

Sensor	Range	Accuracy
Carbon-monoxide concentration sensor	0–2000 ppm	10 ppm
Oxygen-concentration sensor	0%–30%	0.1%
Temperature sensor	0–70 °C	±0.2 K (at 25 °C)
Smoke-concentration sensor	100–5000 ppm	±7%

**Table 2:** Suggestions for handling different danger types and levels.

		Types	Illegal Invasion	Overcrowding	Fire
	Suggestion				
Levels					
Safe			No suggestion.	No suggestion.	No suggestion.
Potentially dangerous			There is a risk of illegal invasion in a certain room, please pay attention to observe the room situation and eliminate potential danger in time.	The number of people in a room has reached x (x is the number of people), and there is a risk of overcrowding. Please pay attention to the gathering of people.	There is a fire risk in a room. Please pay attention to the situation of the room and eliminate the potential danger in time.
Dangerous			A certain room has been invaded, and a certain door and/or window opened abnormally. Please deal with it in time.	The number of people in a certain room has reached x, and the crowd is too dense. Please guide and evacuate.	Fire has broken out in a room. Please rescue immediately.

**Table 3:** Number of samples for different danger types.

		Category	Fire	Illegal Invasion	Overcrowding	Normal	Total
	Number						
Items							
Samples collected			566	567	241	63,426	64,800
Samples used for SVM training and testing			566	567	241	1300	2674

**Table 4:** Types of influencing factors and quantitative results.

Characteristic Variable	Data Type	Maximum	Minimum	Average	Mean-Squared Error
Numerical time	Numerical	0.99	0	0.24	0.40
Temperature (°C)	Numerical	215	25	46.76	45.67
Number of personnel	Numerical	23	0	3.33	4.57
Oxygen concentration (%)	Numerical	23	20	21.52	1.11
Carbon-monoxide concentration (ppm)	Numerical	325	0	53.51	89.0
Smoke concentration (ppm)	Numerical	4957	0	462	1078
Opening and closing of doors	Logical	0-Closing, 1-Opening			
Opening and closing of windows	Logical	0-Closing, 1-Opening			

**Table 5:** Evaluation indices for danger classification using the SVM.

	Category	Fire	Illegal Invasion	Overcrowding
Index				
Accuracy		100%	97.57%	99.25%
Precision		100%	98.15%	97.07%
Recall		100%	99.25%	99.38%

**Table 6:** Numerical types and quantitative results indicating whether danger occurred.

Characteristic Variable	Data Type	Numerical Value and Its Meaning
Whether illegal invasion occurs	Logical	0-No, 1-Yes
Whether fire occurs	Logical	0-No, 1-Yes
Whether overcrowding occurs	Logical	0-No, 1-Yes

## References

[1] Sengan S., Subramaniyaswamy V., Nair S.K., Indragandhi V., Manikandan J., Ravi L. (2020). Enhancing cyber–physical systems with hybrid smart city cyber security architecture for secure public data-smart network. Sustain. Cities. Soc..

[2] Jia M., Komeily A., Wang Y., Srinivasan R.S. (2019). Adopting Internet of Things for the development of smart buildings: A review of enabling technologies and applications. Autom. Constr..

[3] Sung W., Hsiao S. (2020). The application of thermal comfort control based on Smart House System of IoT. Measurement.

[4] Michalec A., Hayes E.T., Longhurst J. (2019). Building smart cities, the just way. A critical review of “smart” and “just” initiatives in Bristol, UK. Sustain. Cities. Soc..

[5] Laufs J., Borrion H., Bradford B. (2020). Security and the smart city: A systematic review. Sustain. Cities. Soc..

[6] Al Dakheel J., Del Pero C., Aste N., Leonforte F. (2020). Smart buildings features and key performance indicators: A review. Sustain. Cities. Soc..

[7] Gramhanssen K., Darby S. (2018). “Home is where the smart is”? Evaluating smart home research and approaches against the concept of home. Energy Res. Soc. Sci..

[8] Hsu H.T., Jong G.J., Chen J.H., Jhe C.G. Improve IoT security system of smart-home by using support vector machine. Proceedings of the 2019 IEEE 4th International Conference on Computer and Communication Systems (ICCCS).

[9] Peng Y., Li S.W., Hu Z.Z. (2015). A self-learning dynamic path planning method for evacuation in large public buildings based on neural networks. Neurocomputing.

[10] Tao F., Liu W., Liu J., Liu X., Liu Q., Qu T., Hu T., Zhang Z., Xiang F., Xu W. (2018). Digital twin and its potential application exploration. Comput. Intergr. Manuf. Syst..

[11] Tao F., Cheng J., Qi Q., Zhang M., Zhang H., Sui F. (2018). Digital twin-driven product design, manufacturing and service with big data. Int. J. Adv. Manuf. Tech..

[12] Zhang H., Ma L., Sun J., Lin H., Thurer M. Digital Twin in Services and Industrial Product Service Systems: Review and Analysis. Proceedings of the 11th CIRP Conference on Industrial Product-Service Systems.

[13] Jones D.M., Snider C., Nassehi A., Yon J., Hicks B.J. (2020). Characterising the Digital Twin: A systematic literature review. CIRP J. Manuf. Sci. Tec..

[14] Papacharalampopoulos A., Giannoulis C., Stavropoulos P., Mourtzis D. (2020). A Digital Twin for Automated Root-Cause Search of Production Alarms Based on KPIs Aggregated from IoT. Appl. Sci..

[15] Papacharalampopoulos A., Stavropoulos P. (2019). Towards a digital twin for thermal processes: Control-centric approach. Procedia CIRP.

[16] Athanasopoulou L., Papacharalampopoulos A., Stavropoulos P. Context awareness system in the use phase of a smart mobility platform: A vision system for a light-weight approach. Proceedings of the 13th CIRP Conference on Intelligent Computation in Manufacturing Engineering, Gulf of Naples.

[17] Liu Z., Bai W., Du X., Zhang A., Xing Z., Jiang A. (2020). Digital Twin-based Safety Evaluation of Prestressed Steel Structure. Adv. Civ. Eng..

[18] Qiuchen L.V., Parlikad A.K., Woodall P., Ranasinghe G.D., Heaton J. Developing a dynamic digital twin at a building level: Using Cambridge campus as case study. Proceedings of the International Conference on Smart Infrastructure and Construction (ICSIC).

[19] Xu D.L., He W., Li S. (2014). Internet of things in industries: A survey. IEEE Trans. Ind. Inf..

[20] Kochovski P., Stankovski V. (2018). Supporting smart construction with dependable edge computing infrastructures and applications. Autom. Constr..

[21] Li N., Becerikgerber B., Krishnamachari B., Soibelman L. (2014). A BIM centered indoor localization algorithm to support building fire emergency response operations. Automat. Construct..

[22] Cheng M., Chiu K., Hsieh Y., Yang I., Chou J., Wu Y. (2017). BIM integrated smart monitoring technique for building fire prevention and disaster relief. Autom. Constr..

[23] Huang F., Liao Z., Wang T., Chen Q., Wu T., Chang C. Intelligent and Disaster Prevention Hard Hat Based on AIOT and Speeches Recognition. Proceedings of the 2019 International Conference on Machine Learning and Cybernetics (ICMLC).

[24] AlHajri M.I., Ali N.T., Shubair R.M. (2018). Classification of indoor environments for IoT applications: A machine learning approach. IEEE Antenn. Wirel. Pr..

[25] Li W., Hu Z., Pei Z., Li S., Chan P.W. (2018). BIM-based integrated delivery technologies for intelligent MEP management in the operation and maintenance phase. Adv. Eng. Softw..

[26] Tang S., Shelden D., Eastman C.M., Pishdadbozorgi P., Gao X. (2019). A review of building information modeling (BIM) and the internet of things (IoT) devices integration: Present status and future trends. Autom. Constr..

[27] Zhang J., Guo J., Xiong H., Liu X., Zhang D. (2019). A Framework for an Intelligent and Personalized Fire Evacuation Management System. Sensors.

[28] Chen X.S., Liu C.C., Wu I.C. (2018). A BIM-based visualization and warning system for fire rescue. Ad. Eng. Inf..

[29] Wu I., Liu C. (2019). A Visual and Persuasive Energy Conservation System Based on BIM and IoT Technology. Sensors.

[30] Zhao L., Liu Z., Mbachu J. (2019). Development of Intelligent Prefabs Using IoT Technology to Improve the Performance of Prefabricated Construction Projects. Sensors.

[31] Cheng J.C., Chen W., Chen K., Wang Q. (2020). Data-driven predictive maintenance planning framework for MEP components based on BIM and IoT using machine learning algorithms. Autom. Constr..

[32] Negri E., Fumagalli L., Macchi M. A review of the roles of digital twin in CPS-based production systems. Proceedings of the 27th International Conference on Flexible Automation and Intelligent Manufacturing, FAIM2017.

[33] Liu S., Bao J., Lu Y., Li J., Lu S., Sun X. Digital Twin Modeling Method Based on Biomimicry for Machining Aerospace Components.

[34] Li W., Rentemeister M., Badeda J., Jöst D., Schulte D., Sauer D.U. (2020). Digital twin for battery systems: Cloud battery management system with online state-of-charge and state-of-health estimation. J. Energy Storage..

[35] Ge S., Zhang F., Wang S., Wang Z. Digital Twin for Smart Coal Mining Workface: Technological Frame and Construction.

[36] Coraddu A., Oneto L., Baldi F., Cipollini F., Atlar M., Savio S. (2019). Data-driven ship digital twin for estimating the speed loss caused by the marine fouling. Ocean Eng..

[37] Kaewunruen S., Lian Q. (2019). Digital twin aided sustainability-based lifecycle management for railway turnout systems. J. Clean. Prod..

[38] Tao F., Liu W., Zhang M., Hu T., Qi Q., Zhang H., Sui F., Wang T., Xu H., Huang Z. (2019). Five dimension digital twin model and its ten applications. Comput. Intergr. Manuf. Syst..

[39] Liu C., Jiang P., Jiang W. (2020). Web-based digital twin modeling and remote control of cyber-physical production systems. Robot. Comput. Integr. Manuf..

[40] Qi Q., Tao F., Hu T., Anwer N., Liu A., Wei Y., Wang L., Nee A.Y.C. Enabling Technologies and Tools for Digital Twin. J. Manuf. Syst..

[41] Boje C., Guerriero A., Kubicki S., Rezgui Y. (2020). Towards a semantic Construction Digital Twin: Directions for future research. Autom. Constr..

[42] Fan C., Zhang C., Yahja A., Mostafavi A. Disaster City Digital Twin: A Vision for Integrating Artificial and Human Intelligence for Disaster Management.

[43] Greif T., Stein N., Flath C.M. (2020). Peeking into the void: Digital twins for construction site logistics. Comput. Ind..

[44] Angjeliu G., Coronelli D., Cardani G. (2020). Development of the simulation model for Digital Twin applications in historical masonry buildings: The integration between numerical and experimental reality. Comput. Struct..

[45] Zhang J., Shan Y., Huang K. (2015). ISEE Smart Home (ISH): Smart video analysis for home security. Neurocomputing.

[46] Qian Y., Liang Y., Li M., Feng G., Shi X. (2014). A resampling ensemble algorithm for classification of imbalance problems. Neurocomputing.

[47] Huang C.L., Wang C.J. (2006). A GA-based feature selection and parameters optimization for support vector Machines. Expert Syst. Appl..

[48] Cherkassky V., Ma Y. (2004). Practical selection of SVM parameters and noise estimation for SVM regression. Neural Netw..

[49] Keerthi S.S., Lin C.J. (2003). Asymptotic behaviors of support vector machines with Gaussian kernel. Neural Comput..

[50] Lin H.T., Lin C.J. (2003). A study on sigmoid kernels for SVM and the training of non-PSD kernels by SMO-type methods. Neural Comput..

[51] Sankaranarayanan S., Mookherji S. (2019). SVM-based traffic data classification for secured IoT-based road signaling system. Int. J. Intell. Inf. Technol..

